# Management of a femoral diaphyseal fracture in a patient with Klippel-Trenaunay-Weber syndrome: a case report

**DOI:** 10.4076/1757-1626-2-8852

**Published:** 2009-08-26

**Authors:** Efstathios Tsaridis, Efthimios Papasoulis, Nikolaos Manidakis, Ioannis Koutroumpas, Savvas Lykoudis, Athanasios Banos, Savvas Sarikloglou

**Affiliations:** Department of Orthopaedics, General Hospital of Kavala62, Amerikanikou Eruthrou, Stavrou Street, 65201 KavalaGreece

## Abstract

**Introduction:**

Klippel-Trenaunay-Weber syndrome is a rare congenital disorder of the peripheral vascular system that is characterized by haemangiomas, soft tissue and/or osseous hypertrophy, venous and lymphatic anomalies as well as arterio-venous malformations. To our knowledge there are no documented cases of surgical fracture management in such patients.

**Case presentation:**

We present the case of a 42-year-old female patient previously diagnosed with Klippel-Trenaunay-Weber syndrome, who had sustained a left femoral shaft fracture. She was treated with a closed, locked intramedullary nailing procedure. The nail was peripherally locked free-hand with a single screw due to the increased vascularity and intraoperative haemorrhage of the area. The patient was transfused with 7 units of blood intra-operatively and was subsequently transferred to the Intensive Care Unit where 3 more units of blood were transfused. Her post-operative course was uneventful. One year following the operation the fracture had united and the patient remained well.

**Conclusion:**

The surgical management of long bone fractures in patients with such pathology is associated with increased intra and post-operative risk, mainly due to vascular complications. It is therefore mandatory that high dependency facilities and sufficient quantities of blood products are available prior to the procedure. A less invasive fixation method should be used when possible.

## Introduction

Klippel-Trenaunay syndrome (nevus vasculosus hypertrophicus) is a rare congenital disorder of the vascular system and is characterized by the following triad of clinical signs. a. Haemangiomas due to cutaneous capillary dysplasias b. Soft tissue and/or bone hypertrophy c. venous and lymphatic anomalies [1]. Diagnosis requires that at least two are present [2]. When arteriovenous malformations co-exist Klippel-Trenaunay-Weber syndrome (or Parkes-Weber) can be diagnosed [3].

It would be expected that the surgical management of long bone fractures in patients with Klippel-Trenaunay-Weber syndrome is associated with intra and post-operative difficulties due to the existing vascular malformations, however no published reports have been found in the literature.

In the herein study we present the management of a femoral diaphyseal fracture with intramedullary nailing in a patient suffering with such syndrome.

## Case presentation

**Figure 1.**
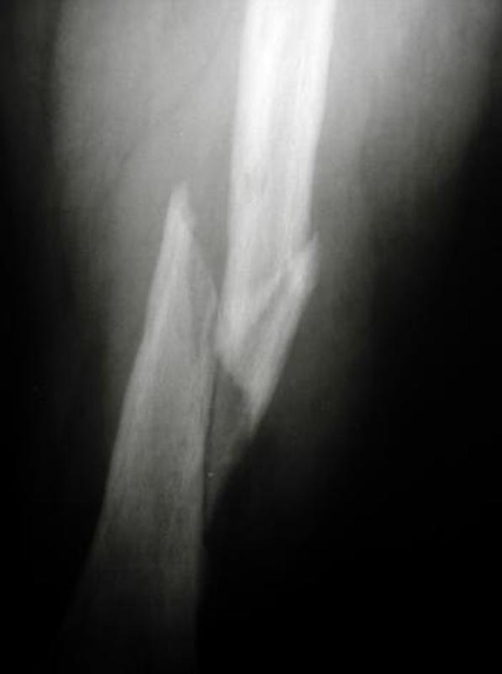
Radiograph at presentation showing the femoral diaphyseal fracture.

**Figure 2.**
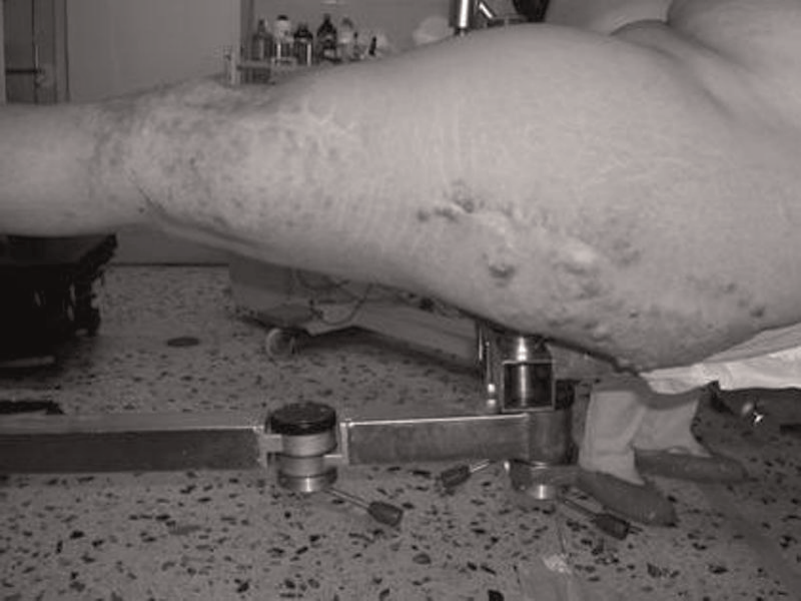
Intra-operative photograph showing multiple venous varicosities affecting the entry point as well as the peripheral locking screw sites of the intramedullary nail.

**Figure 3.**
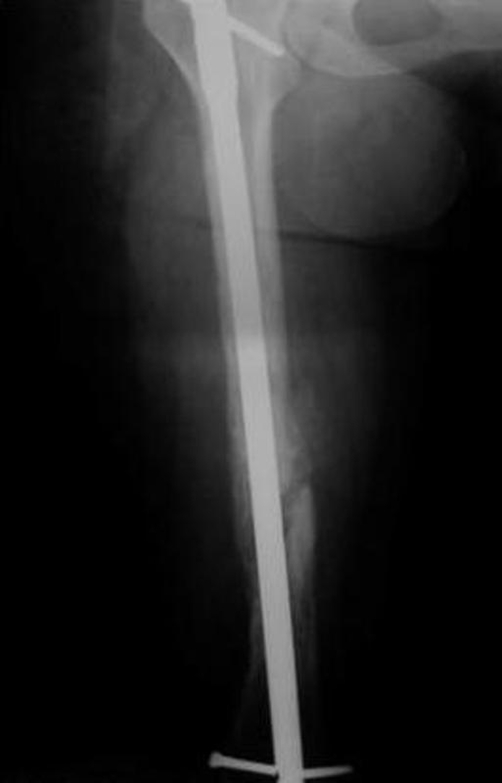
Post-operative radiograph showing union at the fracture site.

A 42-year-old Caucasian female patient of Greek nationality, previously diagnosed with Klippel-Trenaunay-Weber syndrome sustained a left femoral shaft fracture following a road traffic accident (Figure 1). Her past medical history included three vascular surgical interventions in the form of varicosity and malformation resection in another institution. As a result of her condition she presented with arterio-venous malformations, leg length discrepancy of 1.5 cm, and multiple venous varicosities on the lateral aspect of the left lower limb affecting the entry point as well as the peripheral locking screw sites of the intramedullary nail (Figure 2). The reduction of the fracture was performed by closed means and a Russell-Taylor nail was advanced down the femoral shaft. Owing to the vascular malformations the procedure was particularly hemorrhagic. The nail was peripherally locked using a free-hand technique, with a single screw due to the increased vascularity of the lateral aspect of the distal femoral area. The patient was transfused with 7 units of blood intra-operatively and was subsequently transferred to the Intensive Care Unit where 3 more units of blood were transfused. Her post-operative course was uneventful. Eight weeks following the procedure the peripheral screw was found to have broken on check radiographs, as result of full weight bearing, despite medical advice. On the 20^th^ post-operative week clinical and radiological union had been achieved (Figure 3). At the latest follow-up visit one year following surgery the patient remains fully ambulant and pain-free.

## Discussion

The aetiology of Klippel-Trenaunay-Weber syndrome is unknown. Over the years several causative mechanisms such as defect of the sympathetic ganglia [4], deep venous disorders, venous hypertension and varicosities [5], mesodermic anomalies during embryonic development leading to persistence of microscopic arteriovenous malformations [6], mixed meso and ectodermic dysplasias, as well as genomic mutations [7], have been proposed.

Its incidence is sporadic; however unproven autosomal dominant inheritance has also been reported. It affects both sexes and all races equally. It commonly involves the lower limbs (95% of cases) followed by the upper extremities, head, neck and trunk. It usually involves the lateral aspect of a single limb; however multiple limbs can also be affected. When trunk involvement is present, the disease tends to be confined to the midline.

The vascular anomalies can be divided into two main categories. These are tumours (haemangiomas) and vascular dysmorphias [4,6]. The latter are developmental abnormalities that can be further classified into arterial, venous, lymphatic, capillary, mixed and arteriovenous malformations. The vascular dysmorphia characterizing Parkes-Weber syndrome is of arterio-venous nature and is increasing with age due to increasing haemodynamic changes and leads to vascular dilatation, thrombosis and obstruction. Therefore these artriovenous malformations are of progressive nature and their growth is closely associated with puberty, pregnancy, infections and trauma. Disseminated intravascular coagulation has been reported in a patient with Parkes-Weber syndrome following lower leg fracture treated conservatively [8].

History and clinical examination usually suffice for making a diagnosis. In the setting of pre-operative planning and follow up, imaging methods such as computed tomography, magnetic resonance imaging, coloured Doppler, venography and arteriography can be of great value [9].

The majority of patients with Klippel-Trenaunay syndrome are managed conservatively. Epiphysiodesis is recommended in cases where leg length discrepancy of more than 2 cms is present in a growing child. Symptomatic varicosities or localized arteriovenous malformations can be resected [10,11]. Arteriovenous dysmorphias can also be treated with intravascular surgical techniques [9].

Our patient is an interesting case of Klippel-Trenaunay-Weber syndrome from the orthopaedic point of view. The vascular pathology of the affected thigh necessitated closed fracture management, so that avoiding opening the fracture site would leave the blood supply undisturbed and reduce the chance of catastrophic hemorrhage. This was possible by closed reduction and intramedullary nailing. Despite the closed treatment method used, the intra-operative haemorrhage was quite significant due to varicosities and malformations involving the entry point and peripheral locking screw sites.

## Conclusion

The surgical management of long bone fractures in patients with Klippel-Trenaunay-Weber syndrome is associated with high risk due to increased haemorrage. It requires sound pre-operative planning, surgical technique as well as intra and post operative support.
